# Supplementary material for: Recurrent NOMO1 Gene Deletion Is a Potential Clinical Marker in Early-Onset Colorectal Cancer and Is Involved in the Regulation of Cell Migration
Source: Cancers (Basel). 2022 Aug 20;14(16):4029. doi: 10.3390/cancers14164029 (PMC9406593; doi:10.3390/cancers14164029)
Supplement: Supplementary file 1 [file cancers-14-04029-s001.zip › cancers-1839690-supplementary.pdf]

Supplementary Materials: Recurrent *NOMO1* Gene Deletion is a Potential Clinical Marker in Early-Onset Colorectal Cancer and is Involved in the Regulation of Cell Migration

Jésica Pérez-García, Abel Martel-Martel, Paula García-Vallés, Luis A. Corchete, Juan L. García, Nerea Gestoso-Uzal, Rosario Vidal-Tocino, Óscar Blanco, Lucía Méndez, Manuel Sánchez-Martín, Manuel Fuentes, Ana B. Herrero, Andreana N. Holowatyj, José Perea and Rogelio González-Sarmiento

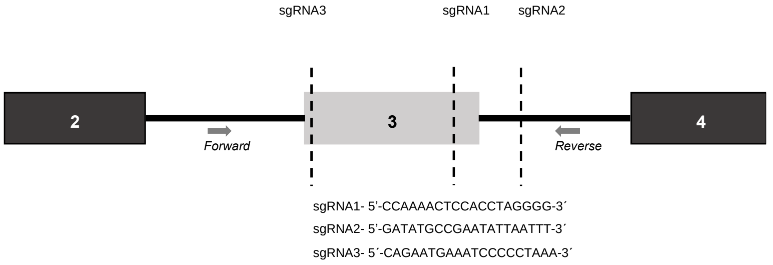

Figure S1. Nucleotide sequences corresponding to each sgRNA used (sgRNA1, sgRNA2, and sgRNA3) and their binding sites.

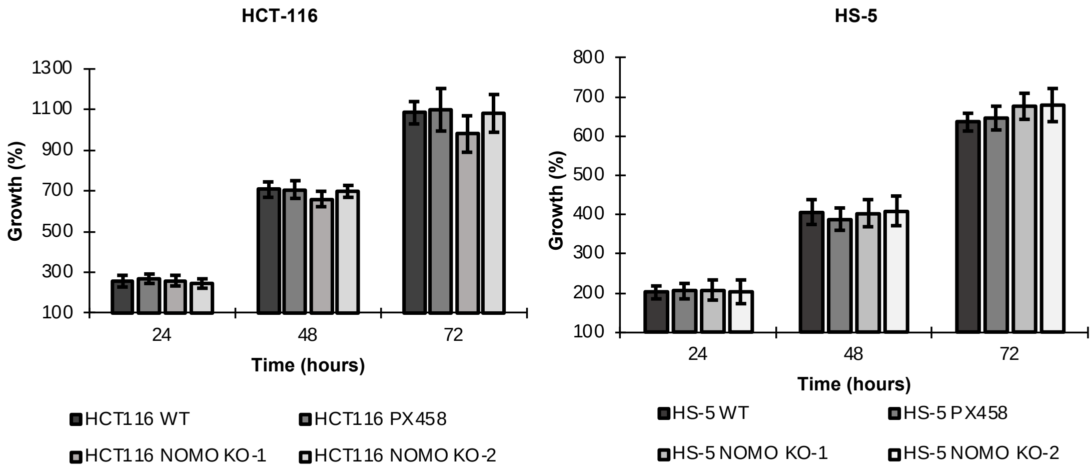

Figure S2. Graphical representation of cell proliferation rates for *NOMO1-KO* and control clones in HCT-116 and HS-5 cell lines. Ratios of cell proliferation were measured at 24, 48, and 72 h. Data are shown as the mean of three replicates ± standard deviation (SD). No statistically significant differences were detected (p-value > 0.05).

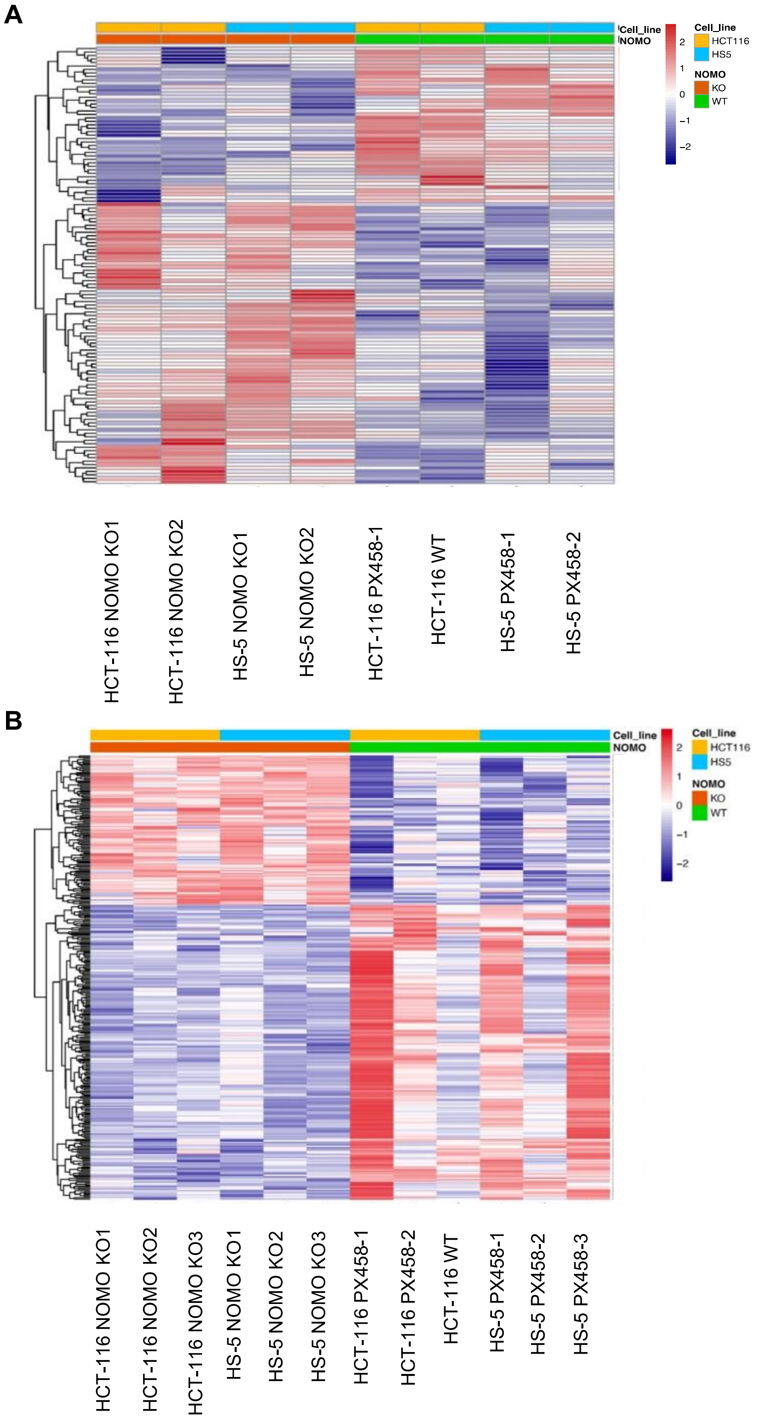

Figure S3. Differentially expressed genes after *NOMO1* inactivation in the two cell lines (HCT-116 and HS-5). **(A)** Heatmap of commonly deregulated genes in the expression microarray assay (FC>1,5; FC<-1,5). **(B)** Heatmap of commonly deregulated genes in the RNA sequencing analysis (FC>1,5; FC<-1,5).

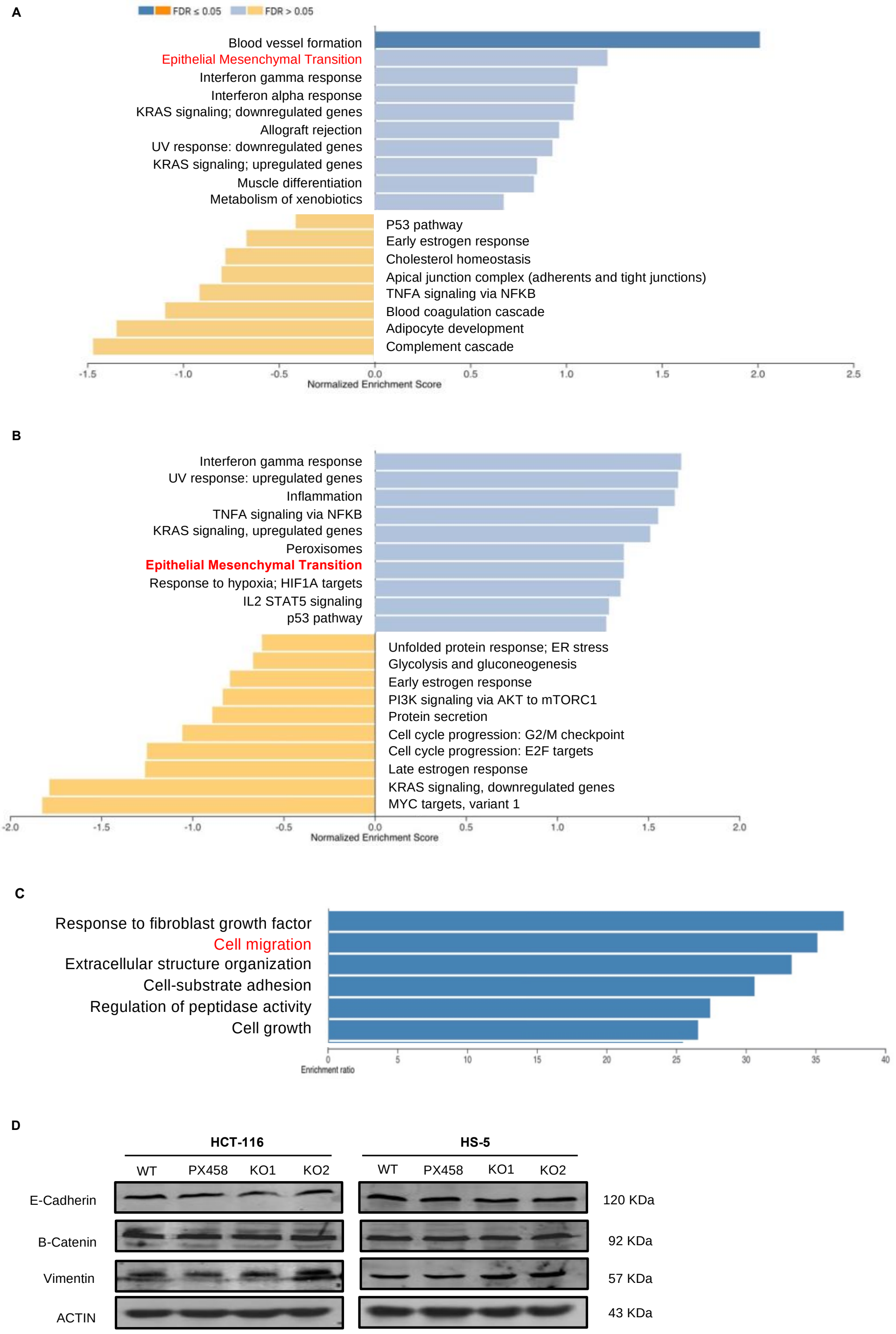

Figure S4. Deregulated biological processes associated with *NOMO1* loss. EMT-associated markers analysis by Western blot. **(A)** Deregulated signaling pathways common to the two cell lines (HCT-116 and HS-5) in *NOMO1-KO* clones, including dysregulated genes in the expression microarray analysis (FC>1,5; FC<-1,5). (**B)** Deregulated signaling pathways in the absence of *NOMO1* common to the two cell lines in *NOMO1-KO* clones, including dysregulated genes in the RNA sequencing analysis (FC>1,5; FC<-1,5). (**C)** Deregulated signaling pathways in the absence of *NOMO1* common to the two *NOMO1-KO* cell lines, including the dysregulated genes that showed to perturb EMT process **(D)** Protein expression analysis of E-Cadherin, B-Catenin, and Vimentin by Western blot in NOMO1-KO and wild-type HCT-116 and HS-5 cell lines.

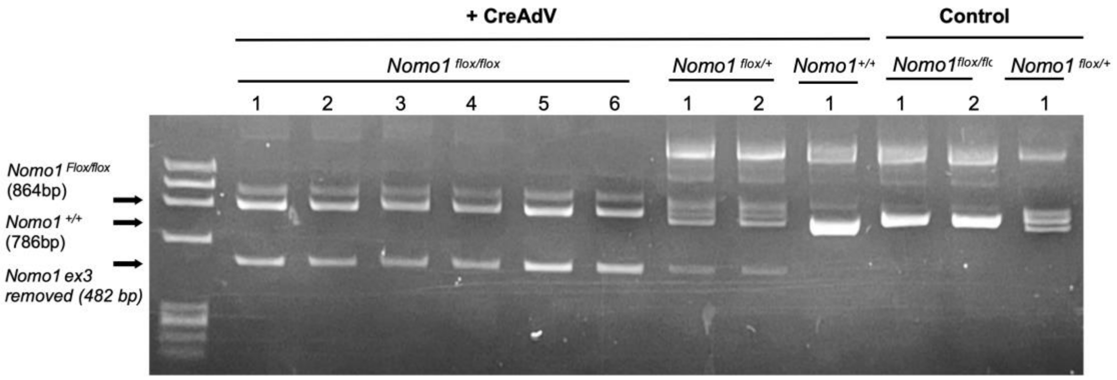

Figure S5. Intestinal genomic DNA was isolated from *Nomo1*$^{flox/flox}$, *Nomo1*$^{flox/+}$, and *Nomo1*$^{+/+}$ mice with and without Cre injection. PCR amplification of *Nomo1* with exon3 deletion detected a 482 bp band due to Cre activity on *Nomo1* floxed locus.

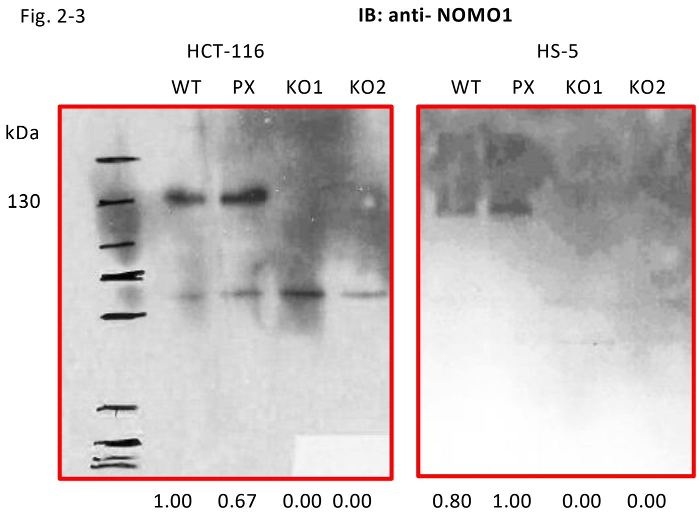

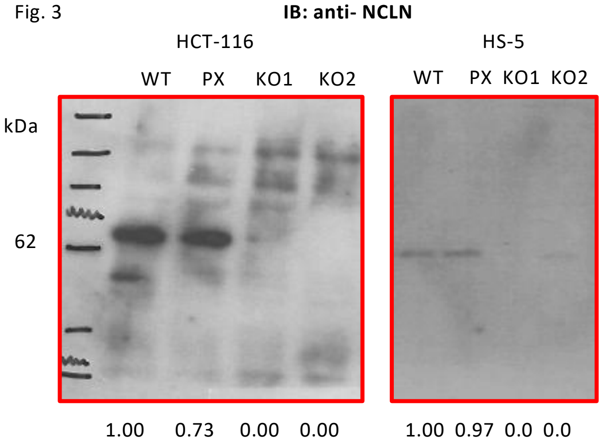

Fig. 3 **IB: anti- TMEM147**

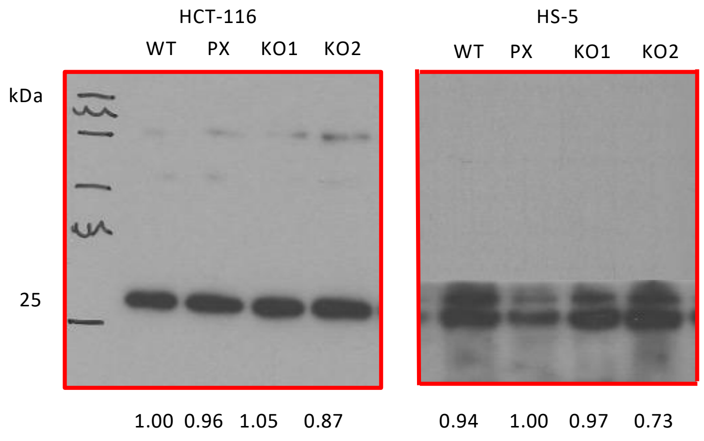

Fig. 2-3 **IB: anti- ACTIN**

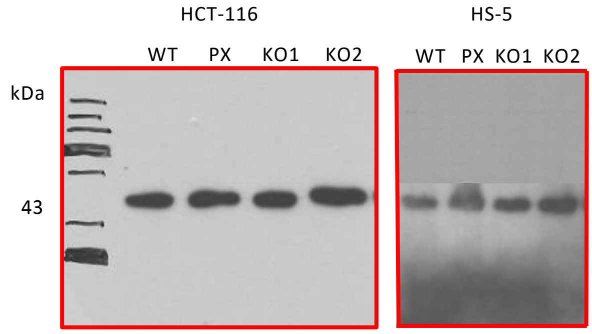

Fig. 4.A **IB: anti- ACTRII**

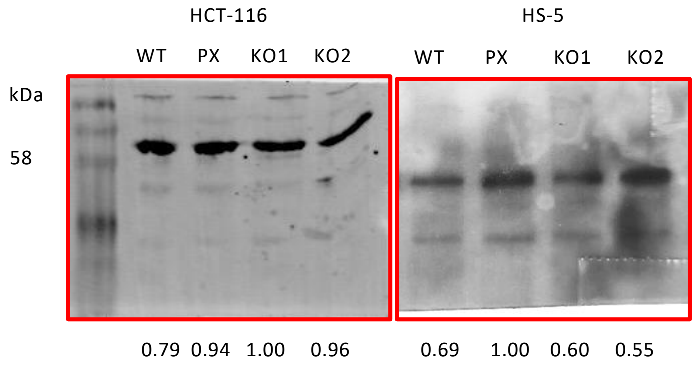

Fig. 4.A **IB: anti- ALK4**

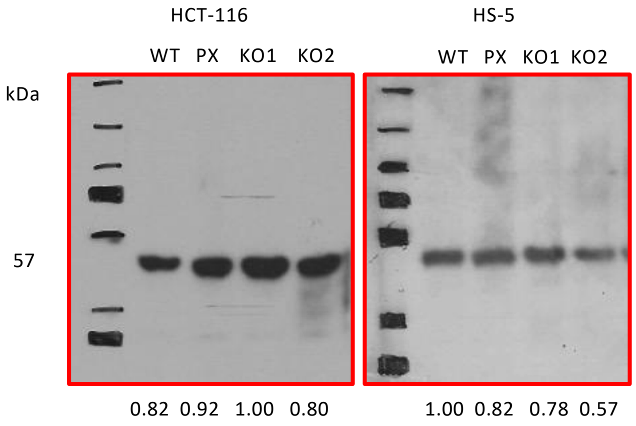

Fig. 4.A **IB: anti- Cripto-1**

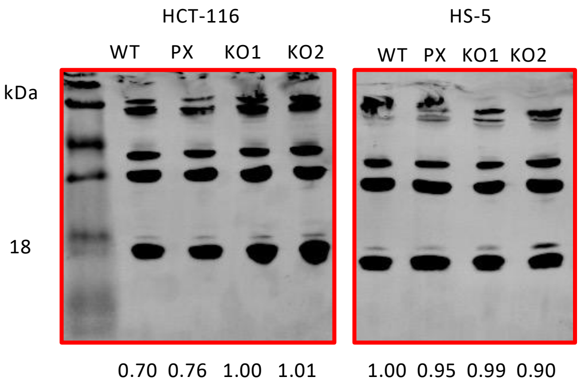

Fig. 4.A **IB: anti- ACTIN**

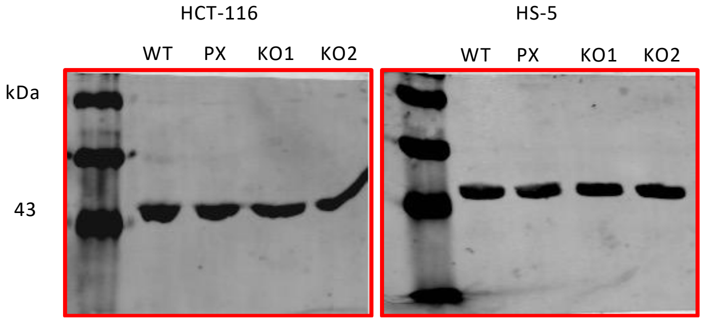

Fig. 4.B **IB: anti- SMAD4**

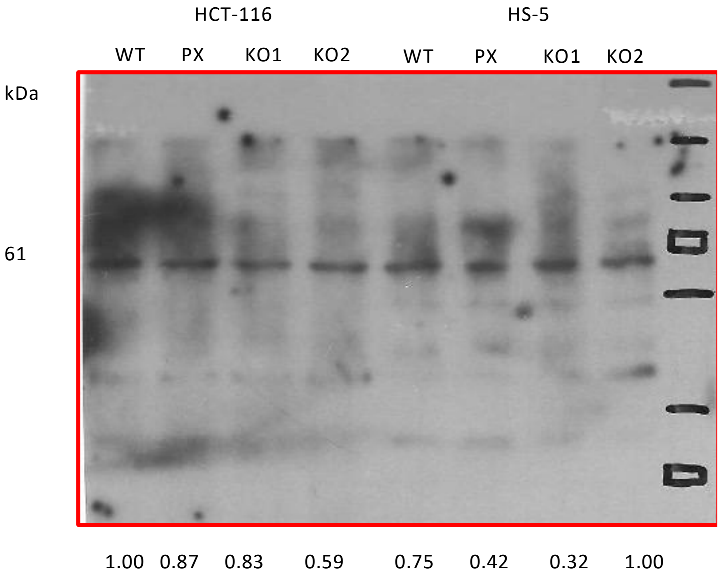

Fig. 4.B

IB: anti- ACTIN

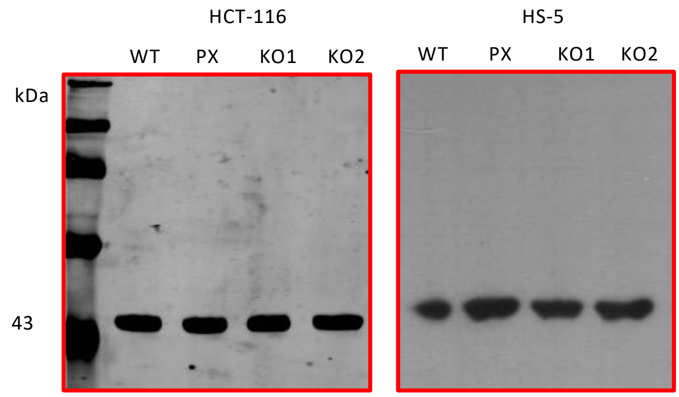

Fig. 4.C

IB: anti- pSMAD2/3

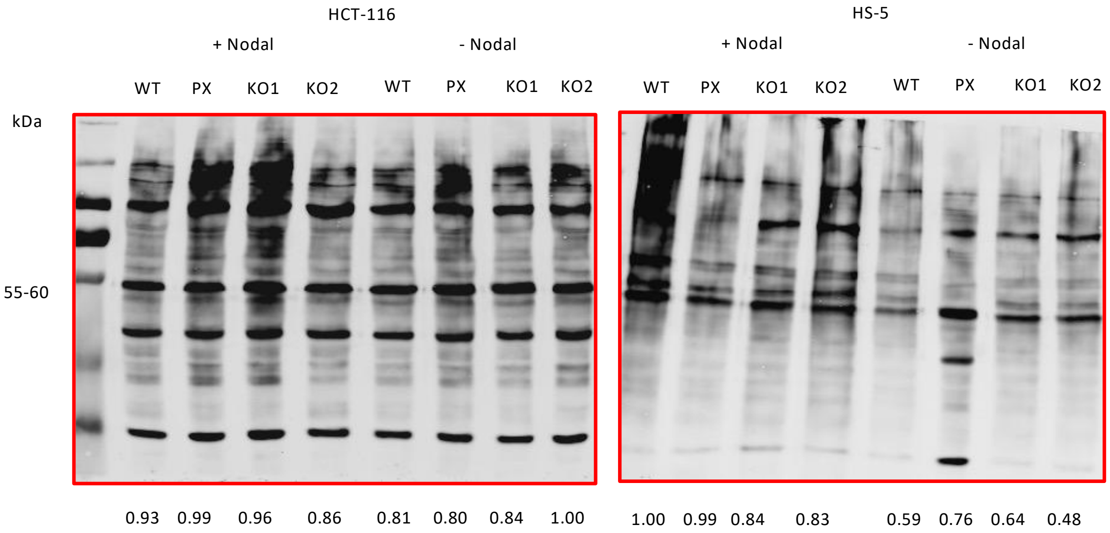

Fig. 4.C

IB: anti- SMAD2/3

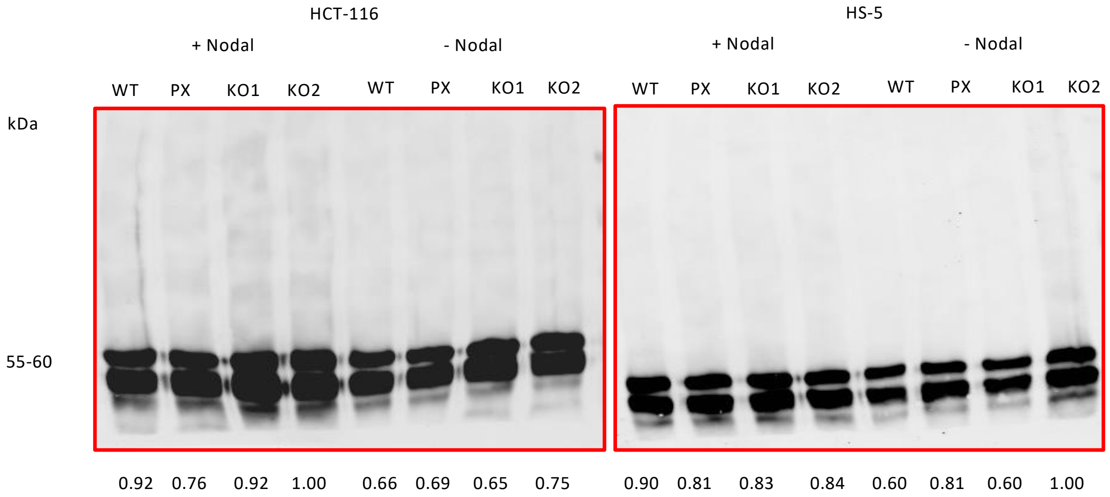

Fig. 4.C

IB: anti- ACTIN

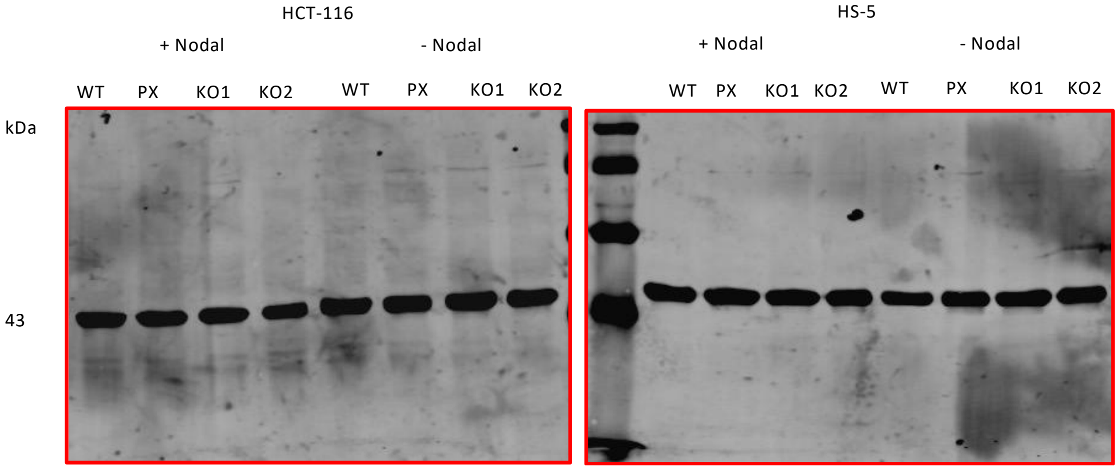

Fig. 5

IB: anti- CTND1

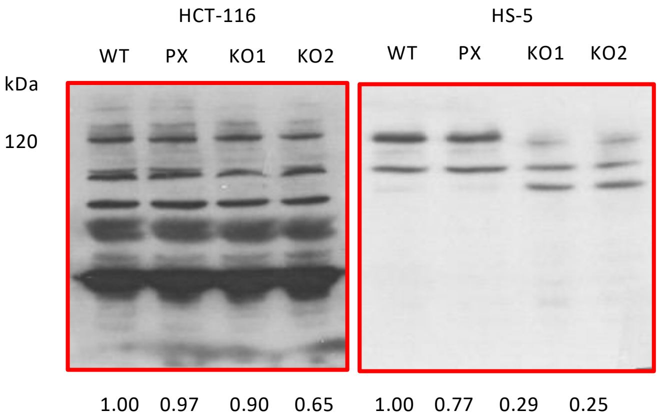

Fig. 5

IB: anti- LMNB1

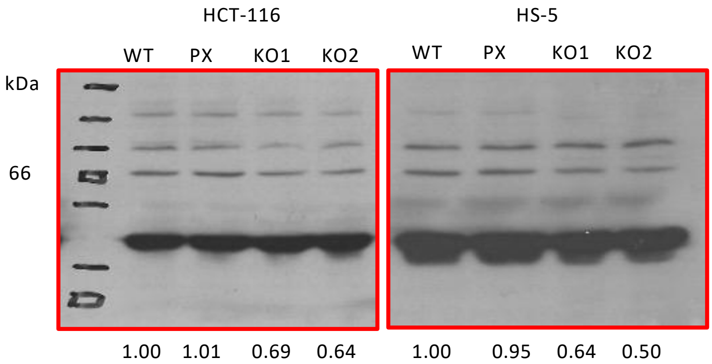

Fig. 5

IB: anti- HMGA1

HCT-116 HS-5

WT PX KO1 KO2 WT PX KO1 KO2

kDa

12

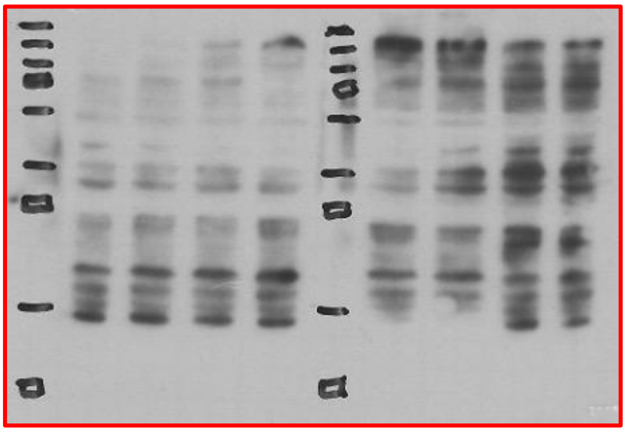

0.79 0.65 1.00 1.04 0.45 0.44 1.00 0.76

Fig. 5

IB: anti- ACTIN

HCT-116 HS-5

WT PX KO1 KO2 WT PX KO1 KO2

kDa

43

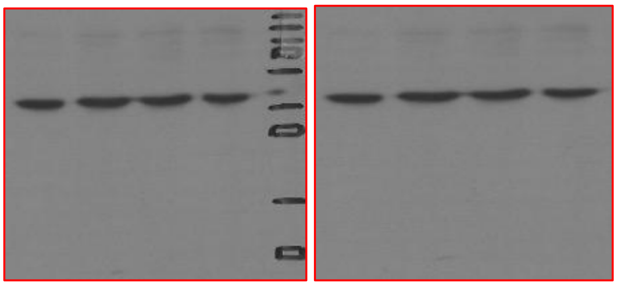

Fig. S4.D

IB: anti- E-Cadherin

HCT-116 HS-5

WT PX KO1 KO2 WT PX KO1 KO2

kDa

120

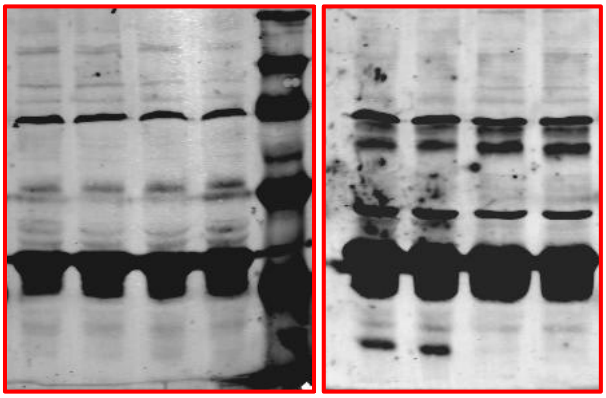

0.93 1.00 0.91 0.76 1.00 0.88 0.93 0.92

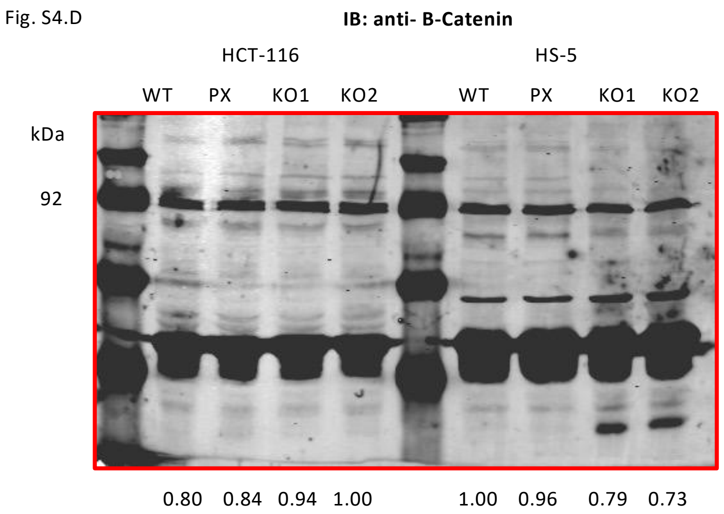

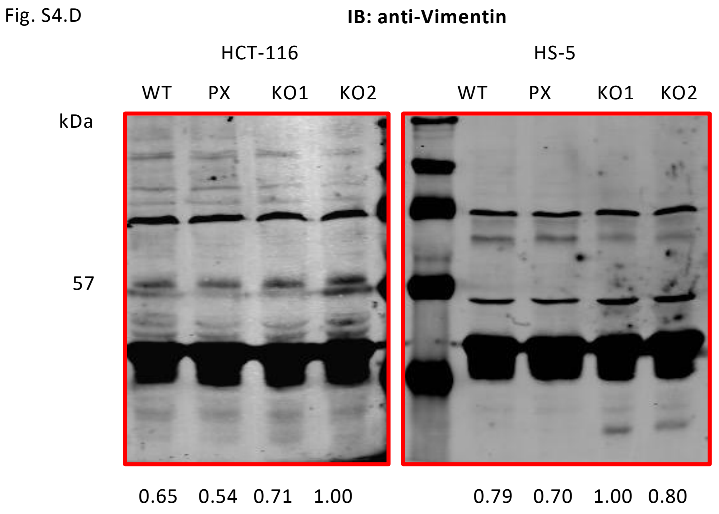

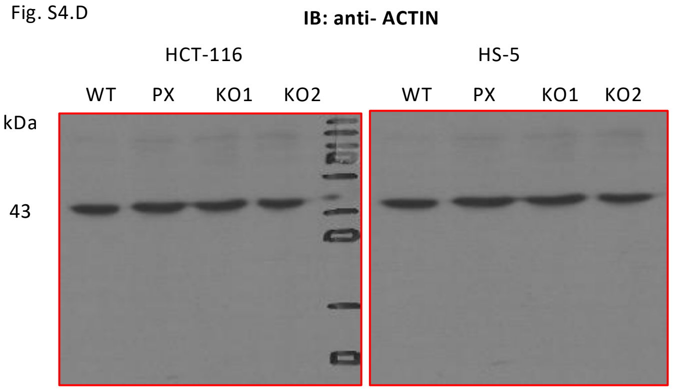

Figure S6. Original uncropped western blots used in this study.

Table S1. Primers used to amplify the specific fragment of each target gene.

GENE	FORWARD PRIMER (5`-3`)	REVERSE PRIMER (3`-5`)
LEMD3 (exon 1)	GCGGCTGCCGGGAGTCTAGACAG	GGTAGGTATGATTGGAGCCCG
NOMO1 (exon 1)	CCGAGCATGCGCCTTAGTTC	ACCAGCATGTCCTCCGAGCC
NOMO1 (exon/intron 4)	AGCTCCATGTGGATGGAGTC	ACGGATGAAGTACAGAGTTC
RBFOX1 (exon 2)	TCTGCATGGTGGCTCCTCAT	AAGGCTGAGCCATTGTGTCA
PKD1 (exon 13)	GTGGAGGGAGGGACGCCAA	ACAGGGAAACCGAGGCTCAGAAA
PDK1 (exon 30)	CTACAGGTGGGTGCCGTAGG	CGCCTTTCCCTCTGGCTGC
STS (exon 5)	TCCTTTACAGGAAGATGAAG	CATTACCAACCTGATAGTTTT
NCLN (exon 4/5)	AGTGACTGGCTGATTGCCA	GCACGCGAACATCCACA
mNomo1 (exon 3)	AGGGAGAAAGAGGATCAGCAG	CCACACAGATTAGGACTCTACT

Table S2. Sequences of oligonucleotides designed as sgRNAs (sgRNA1, sgRNA2, and sgRNA3) and corresponding DNA sequences.

	sgRNA1	sgRNA 2	sgRNA 3
**LOCUS**	5'-CCAAAACTCCACCCTGGGG-3'	5'-GATATGCCGAATATTAATTT-3'	5'-CAGAATGAAATCCCCCTAAA-3'
**Oligonucleotides UP**	5'-caccgCCAAAACTCCACCTAGGGG-3'	5'-caccgGATATGCCGAATATTAATTT-3'	5'-caccgCAGAATGAAATCCCCCTAAA-3'
**Oligonucleotides LOW**	5'-aaacCCCCTAGGGTGGAGTTTTGGc-3'	5'-aaacAAATTAATATTCGGCATATCc-3'	5'-aaacTTTAGGGGGATTTCATTCTGc-3'

Table S3. Antibodies used to study proteins expression by Western blot.

Antibody	Type	Origin	Concentration	Reference	Protein Size (KDa)
**ACTIN**	primary	mouse	1/10000	Sigma Aldrich (A5441)	43
**NOMO1**	primary	goat	1/200	R&D Systems (AF3755)	130
**NCLN**	primary	rabbit	1/500	Sigma Aldrich (SAB1501379)	62
**TMEM147**	primary	rabbit	1/1000	ABCAM(ab97624)	25
**ALK4**	primary	rabbit	1/1000	ABCAM (ab109300)	57
**CRIPTO-1**	primary	rabbit	1/1000	Novus Biologicals(100-1598)	18
**ACTRII-B**	primary	mouse	1/200	Santa Cruz(sc-390977)	58
**P-SMAD2-3**	primary	rabbit	1/1000	ABCAM (ab272332)	55-60
**SMAD2-3**	primary	rabbit	1/1000	ABCAM (ab63672)	55-60
**SMAD4**	primary	mouse	1/1000	Santa Cruz(sc-7966)	61
**E-Cadherin**	primary	mouse	1/1000	Santa Cruz(sc-8426)	120
**Vimentin**	primary	mouse	1/1000	Santa Cruz(sc-6260)	57
**β-Catenin**	primary	mouse	1/1000	BD Biosciences (610154)	92
**CTND1**	primary	rabbit	1/1000	GeneTex (GTX130448)	168
**LMNB1**	primary	rabbit	1/1000	Sigma-Aldrich (ZRB1143)	66
**HMGA1**	primary	rabbit	1/1000	GeneTex (GTX122110)	12
**Anti-Mouse**	secundary	goat	1/10000	GE Healthcare (NXA931)	
**Anti-Rabbit**	secundary	goat	1/10000	Millipore (AP307P)	
**Anti-Goat**	secundary	donkey	1/10000	SCBT (sc-2020)	

Table S4. Microarray dysregulated genes in *NOMO1-KO* clones common to HCT-116 and HS-5 cell lines. Upregulated genes (fold change, FC>1.5) and downregulated genes (FC<-1.5).

Ensembl	Gene	KOvsWT	Ensembl	Gene	KOvsWT
	**Up-regulated**	**FC**		**Down-regulated**	**FC**
ENSG00000139329	*LUM*	2.51	ENSG00000103044	*HAS3*	-2.32
ENSG00000120337	*TNFSF18*	2.35	ENSG00000164484	*TMEM200A*	-2.29
ENSG00000165895	*ARHGAP42*	2.29	ENSG00000154096	*THY1*	-2.28
ENSG00000189058	*APOD*	2.26	ENSG00000133393	*CEP20*	-2.26
ENSG00000170271	*FAXDC2*	2.18	ENSG00000158055	*GRHL3*	-2.12
ENSG00000174501	*ANKRD36C*	2.17	ENSG00000132170	*PPARG*	-2.11
ENSG00000133110	*POSTN*	2.14	ENSG00000135549	*PKIB*	-2.08
ENSG00000003436	*TFPI*	2.06	ENSG00000002745	*WNT16*	-1.98
ENSG00000109452	*INPP4B*	2.03	ENSG00000196954	*CASP4*	-1.84
ENSG00000038427	*VCAN*	2.02	ENSG00000103222	*ABCC1*	-1.81
ENSG00000136040	*PLXNC1*	1.91	ENSG00000101441	*CST4*	-1.76
ENSG00000105855	*ITGB8*	1.89	ENSG00000115339	*GALNT3*	-1.75
ENSG00000164342	*TLR3*	1.87	ENSG00000156804	*FBXO32*	-1.73
ENSG00000116741	*RGS2*	1.86	ENSG00000074416	*MGLL*	-1.72
ENSG00000146457	*WTAP*	1.84	ENSG00000149968	*MMP3*	-1.72
ENSG00000137463	*MGARP*	1.81	ENSG00000175602	*CCDC85B*	-1.72
ENSG00000169429	*CXCL8*	1.81	ENSG00000072864	*NDE1*	-1.71
ENSG00000243137	*PSG4*	1.80	ENSG00000184731	*FAM110C*	-1.70
ENSG00000117586	*TNFSF4*	1.79	ENSG00000113070	*HBEGF*	-1.69
ENSG00000174099	*MSRB3*	1.79	ENSG00000050344	*NFE2L3*	-1.68
ENSG00000122420	*PTGFR*	1.79	ENSG00000100055	*CYTH4*	-1.68
ENSG00000092969	*TGFB2*	1.79	ENSG00000183682	*BMP8A*	-1.66
ENSG00000104313	*EYA1*	1.78	ENSG00000187957	*DNER*	-1.64
ENSG00000265972	*TXNIP*	1.78	ENSG00000185760	*KCNQ5*	-1.64
ENSG00000163661	*PTX3*	1.78	ENSG00000152766	*ANKRD22*	-1.63
ENSG00000177494	*ZBED2*	1.77	ENSG00000235961	*PNMA6A*	-1.62
ENSG00000166482	*MFAP4*	1.76	ENSG00000135374	*ELF5*	-1.62
ENSG00000157110	*RBPMS*	1.74	ENSG00000164300	*SERINC5*	-1.61
ENSG00000189184	*PCDH18*	1.72	ENSG00000106366	*SERPINE1*	-1.61
ENSG00000141574	*SECTM1*	1.72	ENSG00000147394	*ZNF185*	-1.61
ENSG00000164176	*EDIL3*	1.71	ENSG00000170465	*KRT6C*	-1.60
ENSG00000006468	*ETV1*	1.69	ENSG00000125266	*EFNB2*	-1.60
ENSG00000148700	*ADD3*	1.68	ENSG00000165474	*GJB2*	-1.59
ENSG00000135976	*ANKRD36*	1.68	ENSG00000135373	*EHF*	-1.58
ENSG00000138587	*MNS1*	1.67	ENSG00000155066	*PROM2*	-1.56
ENSG00000123609	*NMI*	1.67	ENSG00000110031	*LPXN*	-1.56
ENSG00000134824	*FADS2*	1.66	ENSG00000119866	*BCL11A*	-1.55
ENSG00000203727	*SAMD5*	1.66	ENSG00000144821	*MYH15*	-1.55
ENSG00000053254	*FOXN3*	1.66	ENSG00000149294	*NCAM1*	-1.54
ENSG00000164292	*RHOBTB3*	1.65	ENSG00000167306	*MYO5B*	-1.53
ENSG00000172296	*SPTLC3*	1.65	ENSG00000166211	*SPIC*	-1.52
ENSG00000239467	*0*	1.65	ENSG00000005884	*ITGA3*	-1.52
ENSG00000129038	*LOXL1*	1.64	ENSG00000178445	*GLDC*	-1.52
ENSG00000184500	*PROS1*	1.63	ENSG00000150201	*FXYD4*	-1.51
ENSG00000176697	*BDNF*	1.63	ENSG00000197208	*SLC22A4*	-1.51
ENSG00000164463	*CREBRF*	1.63			
ENSG00000152402	*GUCY1A2*	1.62			
ENSG00000104213	*PDGFRL*	1.62			
ENSG00000143248	*RGS5*	1.61			
ENSG00000183807	*FAM162B*	1.60			
ENSG00000139112	*GABARAPL1*	1.60			
ENSG00000203965	*EFCAB7*	1.60			
ENSG00000196126	*HLA-DRB1*	1.60			
ENSG00000102359	*SRPX2*	1.58			

ENSG00000009413	*REV3L*	1.58
ENSG00000104368	*PLAT*	1.57
ENSG00000242221	*PSG2*	1.56
ENSG00000139625	*MAP3K12*	1.56
ENSG00000138821	*SLC39A8*	1.56
ENSG00000089041	*P2RX7*	1.56
ENSG00000137941	*TTLL7*	1.55
ENSG00000271147	*ARMCX5-GPRASP2*	1.54
ENSG00000000971	*CFH*	1.54
ENSG00000079257	*LXN*	1.54
ENSG00000137752	*CASP1*	1.54
ENSG00000187601	*MAGEH1*	1.53
ENSG00000111799	*COL12A1*	1.52
ENSG00000169126	*ODAD2*	1.52
ENSG00000179818	*PCBP1-AS1*	1.52
ENSG00000077942	*FBLN1*	1.51
ENSG00000152422	*XRCC4*	1.51
ENSG00000124249	*KCNK15*	1.51
ENSG00000106701	*FSD1L*	1.51
ENSG00000021355	*SERPINB1*	1.51
ENSG00000164125	*GASK1B*	1.51
ENSG00000131620	*ANO1*	1.51
ENSG00000164308	*ERAP2*	1.51
ENSG00000125730	*C3*	1.51
ENSG00000170456	*DENND5B*	1.50
ENSG00000151491	*EPS8*	1.50
ENSG00000168876	*ANKRD49*	1.50

Table S5. RNA sequencing dysregulated genes in *NOMO1-KO* clones common to HCT-116 and HS-5 cell lines. Upregulated genes (fold change, FC>1.5) and downregulated genes (FC<-1.5).

Ensembl	Gene	KO/WT	Ensembl	Gene	KO/WT
	**Up-regulated**	**FC**		**Down-regulated**	**FC**
ENSG00000198768	*APCDD1L*	9.39	ENSG00000185164	*NOMO2*	-9.39
ENSG00000115008	*IL1A*	4.73	ENSG00000103226	*NOMO3*	-7.61
ENSG00000113721	*PDGFRB*	3.60	ENSG00000103512	*NOMO1*	-6.95
ENSG00000162645	*GBP2*	3.42	ENSG00000264462	*MIR3648-2*	-6.42
ENSG00000249992	*TMEM158*	2.64	ENSG00000058668	*ATP2B4*	-3.55
ENSG00000179862	*CITED4*	2.51	ENSG00000167978	*SRRM2*	-3.25
ENSG00000166924	*NYAP1*	2.44	ENSG00000212907	*MT-ND4L*	-2.91
ENSG00000167779	*IGFBP6*	2.43	ENSG00000176890	*TYMS*	-2.90
ENSG00000108176	*DNAJC12*	2.40	ENSG00000137801	*THBS1*	-2.68
ENSG00000213442	*RPL18AP3*	2.40	ENSG00000198899	*MT-ATP6*	-2.66
ENSG00000136327	*NKX2-8*	2.33	ENSG00000165121	*0*	-2.54
ENSG00000184986	*TMEM121*	2.30	ENSG00000273373	*0*	-2.54
ENSG00000226525	*RPS7P10*	2.22	ENSG00000198727	*MT-CYB*	-2.53
ENSG00000106333	*PCOLCE*	2.17	ENSG00000143126	*CELSR2*	-2.53
ENSG00000184005	*ST6GALNAC3*	2.14	ENSG00000198886	*MT-ND4*	-2.51
ENSG00000181804	*SLC9A9*	2.13	ENSG00000148773	*MKI67*	-2.44
ENSG00000090238	*YPEL3*	2.08	ENSG00000197943	*PLCG2*	-2.41
ENSG00000236552	*RPL13AP5*	2.04	ENSG00000198763	*MT-ND2*	-2.40
ENSG00000125726	*CD70*	2.04	ENSG00000124222	*STX16*	-2.39
ENSG00000107821	*KAZALD1*	2.02	ENSG00000118523	*CCN2*	-2.38
ENSG00000006118	*TMEM132A*	2.00	ENSG00000096746	*HNRNPH3*	-2.37
ENSG00000099953	*MMP11*	1.96	ENSG00000245532	*NEAT1*	-2.36
ENSG00000104419	*NDRG1*	1.96	ENSG00000080824	*HSP90AA1*	-2.35
ENSG00000104856	*RELB*	1.96	ENSG00000124191	*TOX2*	-2.33

ENSG00000185522	*LMNTD2*	1.96	ENSG00000253626	*EIF5AL1*	-2.31
ENSG00000170955	*CAVIN3*	1.95	ENSG00000170144	*HNRNPA3*	-2.30
ENSG00000163346	*PBXIP1*	1.94	ENSG00000106537	*TSPAN13*	-2.29
ENSG00000003436	*TFPI*	1.94	ENSG00000178966	*RMI1*	-2.28
ENSG00000034053	*APBA2*	1.93	ENSG00000153815	*CMIP*	-2.27
ENSG00000137767	*SQOR*	1.92	ENSG00000231025	*#N/A*	-2.24
ENSG00000135736	*CCDC102A*	1.92	ENSG00000013619	*MAMLD1*	-2.23
ENSG00000240771	*ARHGEF25*	1.90	ENSG00000169564	*PCBP1*	-2.21
ENSG00000142173	*COL6A2*	1.88	ENSG00000171262	*FAM98B*	-2.20
ENSG00000182809	*CRIP2*	1.87	ENSG00000135213	*#N/A*	-2.20
ENSG00000130707	*ASS1*	1.87	ENSG00000068650	*ATP11A*	-2.19
ENSG00000137193	*PIM1*	1.86	ENSG00000183495	*EP400*	-2.19
ENSG00000196878	*LAMB3*	1.86	ENSG00000122566	*HNRNPA2B1*	-2.18
ENSG00000125089	*SH3TC1*	1.83	ENSG00000251562	*MALAT1*	-2.16
ENSG00000197380	*DACT3*	1.83	ENSG00000167548	*KMT2D*	-2.16
ENSG00000131171	*SH3BGRL*	1.83	ENSG00000166845	*C18orf54*	-2.16
ENSG00000100906	*NFKBIA*	1.83	ENSG00000236438	*FAM157A*	-2.15
ENSG00000171798	*KNDC1*	1.79	ENSG00000237438	*CECR7*	-2.14
ENSG00000249264	*EEF1A1P9*	1.78	ENSG00000092969	*TGFB2*	-2.14
ENSG00000142156	*COL6A1*	1.77	ENSG00000159216	*RUNX1*	-2.14
ENSG00000168140	*VASN*	1.76	ENSG00000105649	*RAB3A*	-2.13
ENSG00000145901	*TNIP1*	1.76	ENSG00000234231	*ANAPC1P4*	-2.13
ENSG00000184500	*PROS1*	1.74	ENSG00000089280	*FUS*	-2.13
ENSG00000129757	*CDKN1C*	1.73	ENSG00000158417	*EIF5B*	-2.12
ENSG00000112715	*VEGFA*	1.72	ENSG00000126653	*NSRP1*	-2.11
ENSG00000202515	*VTRNA1-3*	1.71	ENSG00000196935	*SRGAP1*	-2.11
ENSG00000158828	*PINK1*	1.71	ENSG00000102908	*NFAT5*	-2.11
ENSG00000133216	*EPHB2*	1.70	ENSG00000065833	*ME1*	-2.10
ENSG00000104805	*NUCB1*	1.70	ENSG00000156504	*PABIR2*	-2.08
ENSG00000213465	*ARL2*	1.68	ENSG00000085872	*CHERP*	-2.07
ENSG00000230202	*0*	1.68	ENSG00000198888	*MT-ND1*	-2.07
ENSG00000181524	*RPL24P4*	1.67	ENSG00000198646	*NCOA6*	-2.07
ENSG00000103152	*MPG*	1.67	ENSG00000141076	*UTP4*	-2.05
ENSG00000171680	*PLEKHG5*	1.66	ENSG00000136111	*TBC1D4*	-2.04
ENSG00000171223	*JUNB*	1.66	ENSG00000198786	*MT-ND5*	-2.03
ENSG00000105048	*TNNT1*	1.66	ENSG00000179588	*ZFPM1*	-2.03
ENSG00000200312	*RN7SKP255*	1.66	ENSG00000254469	*0*	-2.02
ENSG00000146242	*TPBG*	1.64	ENSG00000048991	*R3HDM1*	-2.02
ENSG00000196205	*EEF1A1P5*	1.64	ENSG00000127124	*HIVEP3*	-2.02
ENSG00000165886	*UBTD1*	1.64	ENSG00000005339	*CREBBP*	-2.02
ENSG00000164932	*CTHRC1*	1.64	ENSG00000196374	*#N/A*	-2.01
ENSG00000143320	*CRABP2*	1.63	ENSG00000180385	*EMC3-AS1*	-2.01
ENSG00000030582	*GRN*	1.63	ENSG00000168488	*ATXN2L*	-2.00
ENSG00000042493	*CAPG*	1.62	ENSG00000196756	*SNHG17*	-2.00
ENSG00000236104	*ZBTB22*	1.62	ENSG00000067082	*KLF6*	-1.98
ENSG00000163702	*IL17RC*	1.62	ENSG00000013588	*GPRC5A*	-1.98
ENSG00000135919	*SERPINE2*	1.62	ENSG00000166436	*TRIM66*	-1.97
ENSG00000184232	*OAF*	1.61	ENSG00000198804	*MT-CO1*	-1.97
ENSG00000204592	*HLA-E*	1.61	ENSG00000198712	*MT-CO2*	-1.95
ENSG00000109861	*CTSC*	1.61	ENSG00000102287	*GABRE*	-1.95
ENSG00000234745	*HLA-B*	1.61	ENSG00000129173	*E2F8*	-1.94
ENSG00000213859	*KCTD11*	1.60	ENSG00000198556	*ZNF789*	-1.94
ENSG00000013364	*MVP*	1.59	ENSG00000116698	*SMG7*	-1.93
ENSG00000125637	*PSD4*	1.59	ENSG00000101333	*PLCB4*	-1.93
ENSG00000218426	*0*	1.58	ENSG00000159110	*IFNAR2*	-1.93
ENSG00000196208	*GREB1*	1.58	ENSG00000145819	*ARHGAP26*	-1.92
ENSG00000100558	*PLEK2*	1.58	ENSG00000090061	*CCNK*	-1.92
ENSG00000206503	*HLA-A*	1.57	ENSG00000140534	*TICRR*	-1.92
ENSG00000125657	*TNFSF9*	1.57	ENSG00000026508	*CD44*	-1.91

ENSG00000163795	*ZNF513*	1.57
ENSG00000115756	*HPCAL1*	1.56
ENSG00000135124	*P2RX4*	1.56
ENSG00000172889	*EGFL7*	1.56
ENSG00000167552	*TUBA1A*	1.55
ENSG00000161653	*NAGS*	1.55
ENSG00000181104	*F2R*	1.55
ENSG00000011028	*MRC2*	1.55
ENSG00000244313	*0*	1.55
ENSG00000099364	*FBXL19*	1.54
ENSG00000125912	*NCLN*	1.54
ENSG00000013563	*DNASE1L1*	1.54
ENSG00000183405	*#N/A*	1.54
ENSG00000167671	*UBXN6*	1.53
ENSG00000130775	*THEMIS2*	1.52
ENSG00000156587	*UBE2L6*	1.52
ENSG00000101940	*WDR13*	1.52
ENSG00000105419	*MEIS3*	1.51
ENSG00000133247	*KMT5C*	1.51
ENSG00000108679	*LGALS3BP*	1.51
ENSG00000174791	*RIN1*	1.51
ENSG00000126432	*PRDX5*	1.51
ENSG00000114796	*KLHL24*	1.50
ENSG00000154134	*ROBO3*	1.50
ENSG00000204054	*LINC00963*	1.50
ENSG00000116260	*QSOX1*	1.50
ENSG00000105404	*RABAC1*	1.50

ENSG00000159140	*SON*	-1.91
ENSG00000117523	*PRRC2C*	-1.90
ENSG00000165521	*EML5*	-1.90
ENSG00000160201	*U2AF1*	-1.90
ENSG00000139734	*DIAPH3*	-1.88
ENSG00000034152	*MAP2K3*	-1.88
ENSG00000223959	*AFG3L1P*	-1.88
ENSG00000101557	*USP14*	-1.87
ENSG00000133858	*ZFC3H1*	-1.87
ENSG00000138668	*HNRNPD*	-1.87
ENSG00000211459	*MT-RNR1*	-1.87
ENSG00000157741	*UBN2*	-1.87
ENSG00000145029	*NICN1*	-1.85
ENSG00000005100	*DHX33*	-1.85
ENSG00000120685	*PROSER1*	-1.85
ENSG00000153989	*NUS1*	-1.84
ENSG00000178127	*NDUFV2*	-1.84
ENSG00000214425	*LRRC37A4P*	-1.83
ENSG00000264112	*0*	-1.83
ENSG00000239665	*0*	-1.82
ENSG00000126870	*DYNC2I1*	-1.82
ENSG00000181827	*RFX7*	-1.81
ENSG00000213064	*SFT2D2*	-1.81
ENSG00000114316	*USP4*	-1.81
ENSG00000164040	*PGRMC2*	-1.81
ENSG00000146909	*NOM1*	-1.81
ENSG00000197111	*PCBP2*	-1.81
ENSG00000112578	*BYSL*	-1.80
ENSG00000174231	*PRPF8*	-1.80
ENSG00000124164	*VAPB*	-1.80
ENSG00000259758	*#N/A*	-1.79
ENSG00000104881	*PPP1R13L*	-1.79
ENSG00000106144	*CASP2*	-1.79
ENSG00000152818	*UTRN*	-1.78
ENSG00000142875	*PRKACB*	-1.77
ENSG00000226887	*ERVMER34-1*	-1.76
ENSG00000188033	*ZNF490*	-1.75
ENSG00000127947	*PTPN12*	-1.75
ENSG00000168813	*ZNF507*	-1.75
ENSG00000171848	*RRM2*	-1.75
ENSG00000113360	*DROSHA*	-1.75
ENSG00000153317	*ASAP1*	-1.75
ENSG00000140948	*ZCCHC14*	-1.74
ENSG00000101898	*MCTS2P*	-1.74
ENSG00000137822	*TUBGCP4*	-1.73
ENSG00000164190	*NIPBL*	-1.73
ENSG00000187605	*TET3*	-1.73
ENSG00000114790	*ARHGEF26*	-1.73
ENSG00000185697	*MYBL1*	-1.73
ENSG00000136108	*CKAP2*	-1.73
ENSG00000162601	*MYSM1*	-1.73
ENSG00000139133	*ALG10*	-1.72
ENSG00000174501	*ANKRD36C*	-1.72
ENSG00000120948	*TARDBP*	-1.72
ENSG00000113300	*CNOT6*	-1.72
ENSG00000213240	*#N/A*	-1.72
ENSG00000138071	*ACTR2*	-1.71
ENSG00000123200	*ZC3H13*	-1.71

ENSG00000139645	*ANKRD52*	-1.71
ENSG00000060749	*QSER1*	-1.71
ENSG00000142871	*CCN1*	-1.70
ENSG00000120694	*HSPH1*	-1.70
ENSG00000134243	*SORT1*	-1.69
ENSG00000104131	*EIF3J*	-1.69
ENSG00000144028	*SNRNP200*	-1.69
ENSG00000160193	*WDR4*	-1.69
ENSG00000110958	*PTGES3*	-1.68
ENSG00000167881	*SRP68*	-1.68
ENSG00000096060	*FKBP5*	-1.67
ENSG00000051341	*POLQ*	-1.67
ENSG00000136146	*MED4*	-1.67
ENSG00000101126	*ADNP*	-1.67
ENSG00000121774	*KHDRBS1*	-1.66
ENSG00000143919	*CAMKMT*	-1.66
ENSG00000196233	*LCOR*	-1.66
ENSG00000135457	*TFCP2*	-1.65
ENSG00000268205	*0*	-1.65
ENSG00000146676	*PURB*	-1.65
ENSG00000126216	*TUBGCP3*	-1.64
ENSG00000140941	*MAP1LC3B*	-1.64
ENSG00000139514	*SLC7A1*	-1.64
ENSG00000068878	*PSME4*	-1.63
ENSG00000173517	*PEAK1*	-1.63
ENSG00000125863	*MKKS*	-1.63
ENSG00000119596	*YLPM1*	-1.62
ENSG00000111731	*C2CD5*	-1.62
ENSG00000235655	*H3P6*	-1.62
ENSG00000187951	*0*	-1.62
ENSG00000119396	*RAB14*	-1.61
ENSG00000258441	*LINC00641*	-1.61
ENSG00000197170	*PSMD12*	-1.61
ENSG00000103540	*CCP110*	-1.60
ENSG00000196715	*VKORC1L1*	-1.60
ENSG00000188786	*MTF1*	-1.60
ENSG00000078140	*UBE2K*	-1.60
ENSG00000102221	*JADE3*	-1.60
ENSG00000178691	*SUZ12*	-1.60
ENSG00000112699	*GMDS*	-1.59
ENSG00000108848	*LUC7L3*	-1.59
ENSG00000033627	*ATP6V0A1*	-1.58
ENSG00000226380	*0*	-1.58
ENSG00000148840	*PPRC1*	-1.58
ENSG00000008282	*SYPL1*	-1.58
ENSG00000088836	*SLC4A11*	-1.57
ENSG00000087053	*MTMR2*	-1.57
ENSG00000153904	*DDAH1*	-1.57
ENSG00000170448	*NFXL1*	-1.56
ENSG00000115904	*SOS1*	-1.56
ENSG00000112159	*MDN1*	-1.54
ENSG00000083642	*PDS5B*	-1.54
ENSG00000158169	*FANCC*	-1.54
ENSG00000144909	*OSBPL11*	-1.54
ENSG00000187231	*SESTD1*	-1.54
ENSG00000172954	*LCLAT1*	-1.54
ENSG00000196584	*XRCC2*	-1.54
ENSG00000157106	*SMG1*	-1.53
ENSG00000136381	*IREB2*	-1.53

ENSG00000166913	*YWHAB*	-1.53
ENSG00000011021	*CLCN6*	-1.53
ENSG00000152223	*EPG5*	-1.53
ENSG00000166135	*HIF1AN*	-1.52
ENSG00000151233	*GXYLT1*	-1.52
ENSG00000023516	*AKAP11*	-1.52
ENSG00000253352	*TUG1*	-1.52
ENSG00000103404	*USP31*	-1.52
ENSG00000253729	*PRKDC*	-1.51
ENSG00000113810	*SMC4*	-1.51
ENSG00000132953	*XPO4*	-1.51
ENSG00000153574	*RPIA*	-1.50
ENSG00000205765	*C5orf51*	-1.50

Table S6. Dysregulated proteins associated with *NOMO1* loss common to HCT-116 and HS-5 cell lines. Protein expression levels are showed as >1 or <1 for upregulated downregulated proteins, respectively.

UniProt ID	Protein	HCT-116	HS-5
	**Down-regulated**	**KO/WT**	**KO/WT**
O43707	ACTN4	0.895	0.777
O60716	CTND1	0.840	0.582
P08238	HS90B	0.909	0.791
P09429	HMGB1	0.867	0.671
P12429	ANXA3	0.655	0.197
P20700	Lamin-B1	0.760	0.674
P23921	RIR1	0.784	0.494
P26006	Integrin alpha-3	0.517	0.433
P45974	UBP5	0.793	0.661
P69849	NOMO3	0.024	0.017
Q5QJE6	TDIF2	0.556	0.492
Q92522	H1X	0.490	0.642
Q969V3	NCLN	0.059	0.085
Q9H3U1	UN45A	0.607	0.312
Q9NZM1	MYOF	0.682	0.696
Q9Y5X1	SNX9	0.719	0.720
	**Up-regulated**	**KO/WT**	**KO/WT**
Q8IYS2	K2013	2,710	4,593
O94808	GFPT2	2,108	3,677
P06703	S10A6	2,013	3,470
P37268	FDFT	1,993	2,857
P17096	HMGA1	1,985	2,650
P48594	SPB4	1,805	2,549
P57088	TMM33	1,787	1,866
P10301	RRAS	1,471	1,793
Q15738	NSDHL	1,429	1,607
Q06830	PRDX1	1,314	1,444
Q13617	CUL2	1,258	1,404
P00367	DHE3	1,144	1,354
